# Acute Cerebral Ischemia Increases a Set of Brain-Specific miRNAs in Serum Small Extracellular Vesicles

**DOI:** 10.3389/fnmol.2022.874903

**Published:** 2022-04-27

**Authors:** Xin Zhou, Chenxue Xu, Dachong Chao, Zixin Chen, Shuyuan Li, Miaomiao Shi, Yuqiang Pei, Yujuan Dai, Juling Ji, Yuhua Ji, Qiuhong Ji

**Affiliations:** ^1^College of Life Science and Technology, Institute of Immunology, Jinan University, Guangzhou, China; ^2^The First Affiliated Hospital, Jinan University, Guangzhou, China; ^3^Department of Neurology, Affiliated Hospital of Nantong University, Nantong, China; ^4^Department of Pathology, Medical School of Nantong University, Nantong, China; ^5^Key Laboratory of Neuroregeneration of Jiangsu and Ministry of Education, Nantong University, Nantong, China

**Keywords:** ischemic stroke, serum sEVs, miRNAs, RNA-Seq, tMCAO

## Abstract

Small extracellular vesicles (sEVs) miRNAs are promising diagnosis and prognosis biomarkers for ischemic stroke (IS). This study aimed to determine the impact of IS on the serum sEVs miRNA profile of IS patients and a transient middle cerebral artery occlusion (tMCAO) mouse model. Small RNAseq was used to define the serum sEVs miRNA profile in IS patients and healthy controls (HC), and tMCAO mice and sham controls. Among the 1,444 and 1,373 miRNAs identified in human and mouse serum sEVs, the expression of 424 and 37 miRNAs was significantly altered in the IS patients and tMCAO mice, respectively (| Log_2_FC| ≥ 1, *p* < 0.01). Notably, five of the top 25 upregulated miRNAs in IS patients were brain-specific or enriched, including hsa-miR-9-3p, hsa-miR-124-3p, hsa-miR-143-3p, hsa-miR-98-5p, and hsa-miR-93-5p. Upregulation of these four miRNAs was further validated by qPCR. Nine of the 20 upregulated miRNAs in tMCAO mice were also brain-specific or enriched miRNAs. Temporal analysis indicated that the dynamics of mmu-miR-9-5p, mmu-miR-124-3p, mmu-miR-129-5p, and mmu-miR-433-3p were closely correlated with the evolution of ischemic brain injury, as their expression increased at 0.5 days after the onset of ischemia, peaked at day 1 or 3, and returned to normal levels at day 7 and 14. Notably, with the exceptions of mmu-miR-128-3p, the expression of the other eight miRNAs in the mouse serum sEVs was unaffected in the lipopolysaccharide (LPS)-induced neuroinflammation model. Together, in this study, we provided a comprehensive view of the influences of IS on the serum sEVs miRNA profile of IS patients and tMCAO mice and demonstrated the increment of a set of brain-specific miRNAs in serum sEVs after acute cerebral ischemia, which could be promising candidates directly reflecting the ischemic brain injury.

## Introduction

Stroke is a leading cause of death and adult disability worldwide. Approximately 80% are ischemic stroke (IS) (Mortality and Causes of Death, 2016). Although CT (Computed Tomography) and MRI (Magnetic Resonance Imaging) are widely implemented in stroke diagnosis, biomarkers that could facilitate the IS diagnosis, prognosis prediction, and therapeutic evaluation are valuable for improving the clinical treatment of IS patients (Saenger and Christenson, 2010; Karakas and Zeller, 2017).

MiRNAs are a class of endogenous 22 nucleotides non-coding RNAs (Bartel, 2004). In addition to post-transcriptional regulation of gene expression by mediating targeted hydrolysis and translation inhibition, they are detectable in various biological fluids (Weber et al., 2010). Because of their stability and relative tissue specificity, circulating miRNAs are attractive candidates for biomarker research (Creemers et al., 2012; He et al., 2015). Small extracellular vesicles (sEVs), including exosomes, are nanoscale vesicles released by almost all cell types. sEVs are the dominant form of circulating RNA (Johnstone et al., 1989; Théry et al., 2002). Several groups, including us, have recently reported some serum exosomal miRNA candidates for IS diagnosis, including miR-9 and miR-124 (Ji et al., 2016), miR-223 (Chen et al., 2017), miR-422a and miR-125b-2-3p (Li D.B. et al., 2017), miR-21-5p and miR-30a-5p (Wang et al., 2018), and miR-17 family members (Van Kralingen et al., 2019). Serum sEVs contain hundreds of miRNAs (Li X. et al., 2017; Zhao et al., 2020). However, the effects of cerebral ischemia on miRNAs profile of serum sEVs is still unclear.

In this study, using an unbiased small RNAseq, we comprehensively examined the impact of acute cerebral ischemia on the profile of serum sEVs miRNAs in IS patients and a tMCAO mouse model. Besides the profound effects of cerebral ischemia on the serum sEVs miRNA profile, we noticed that the level of a set of brain-specific or enriched miRNAs significantly increased in both IS patients and tMCAO mouse models. Further animal experiments indicated that the temporal expression of these brain-specific miRNAs was closely correlated with the evolution of cerebral ischemia injury. It is worth noting that most of these altered brain-specific miRNAs were unaffected by the LPS-induced neuroinflammation.

## Materials and Methods

### Subject Demographics and Clinical Characteristics

Ischemic stroke patients were recruited from those admitted to the Department of Neurology of the Affiliated Hospital of Nantong University between September 2016 and August 2018. Ischemic stroke was confirmed by either MRI or CT imaging. In addition, experienced neurologists determined the neurological deficits by using the NIHSS. We excluded patients with intracranial hemorrhage and infarction with unknown causes and those with malignant tumors or neurological and psychiatric diseases for all individuals involved in this study. Venous blood samples were collected on admission before any treatment. The average time of enrollment blood draw was 18.5 h. Non-stroke healthy controls (HCs) were recruited from those who underwent an annual medical examination. The demographics and clinical characteristics of the 40 IS patients and 33 HCs are provided in Table 1.

**TABLE 1:** Baseline characteristics of study participants.

Parameter	HCs	IS (11–72 h)	*p* values*
**Age and gender**			
Total, *n*	33	40	
Male, *n* (%)	18 (55)	18 (45)	0.417
Age (y), mean (SD)	63.67 (8.9)	68.1 (11.9)	0.0813
Stroke risk factors, *n* (%)			
Hypertension	17 (51.5)	22 (55)	0.733
Diabetes mellitus	5 (15.2)	8 (20)	0.590
Hyerlipidaemia	6 (18.2)	9 (22.5)	0.594
Smoker	4 (12.1)	5 (12.5)	0.497
History of stroke/TIA	2 (6)	6 (15)	0.058
**Laboratory parameters, mean ± SD**			
Glucose, mmol/L	6.57 ± 1.73	7.14 ± 2.08	0.2133
Platelets, 10^9^/L	210.9 ± 41.06	191.6 ± 55.7	0.1027
Triglycerides, mmol/L	1.19 ± 0.60	1.22 ± 0.88	0.8683
Total cholesterol, mmol/L	4.66 ± 0.82	4.35 ± 0.99	0.1551
HDL, mmol/L	1.33 ± 0.27	1.24 ± 0.37	0.2482
LDL, mmol/L	2.68 ± 0.54	2.46 ± 0.73	0.1553
WBC count, 10^9^/L	5.85 ± 0.96	6.7 ± 2.69	0.0889
RBC count, 10^12^/L	4.71 ± 0.43	4.52 ± 0.47	0.0784
NIHSS score			<0.01
Mean (25th–75th percentile)	0 (0)	10 (6.75-14)	
Infarct volume			
Mean (25th–75th percentile), mL	n/a	12.70 (3.04-25.87)	<0.01

*For Continuous variables, a Wilcoxon test was used to assess differences, while for categorical variables, a Fisher’s exact test was used.

*HDL, high-density lipoprotein; LDL, low-density lipoprotein; n/a, not available, RBC, red blood cell; TIA, transient ischemic attack; WBC, white blood cell.*

### Transient Middle Cerebral Artery Occlusion Model

Male wild-type C57BL/6 mice (8–9 weeks) were purchased from Guangdong medical laboratory animal center and used in all experiments within 1 week of arrival. Anesthesia was induced with 4% isoflurane and maintained on 2% isoflurane in the air. Transient cerebral ischemia was conducted by occlusion of the left middle cerebral artery with a monofilament suture (602356, Doccol Corp., Redlands, CA, United States) for 60 min. Rectal body temperature was maintained at 37 ± 0.5°C during surgery. Sham-operated mice were subjected to all surgical procedures except suture advancement. One day after ischemia, the animals were euthanized with an overdose of isoflurane, and the left ventricular puncture drew blood.

### 2,3,5-Triphenyltetrazolium Chloride Staining and Measurement of Infarct Volume

For TTC staining, the brain was sectioned coronally at 1-mm thickness. The slices were immersed in 1% 2,3,5-triphenyltetrazolium chloride (TTC, Sigma Aldrich, St. Louis, MO, United States) in saline for 30 min at 37°C and then fixed with 4% paraformaldehyde. Infarction volume was measured on six coronal sections using ImageJ.^1^ The indirect infarct area, in which the intact area of the ipsilateral hemisphere was subtracted from the area of the contralateral hemisphere, was calculated. The ischemic volume was presented as the percentage of infarct volume of the contralateral hemisphere.

### Lipopolysaccharide Challenge

Adult male C57BL/6 mice were randomized to receive intraperitoneal injections of either 1 mg/kg lipopolysaccharide (LPS) (*Escherichia coli* 0556:B5, Sigma Aldrich, St. Louis, MO, United States) or phosphate-buffered saline (PBS). The animals were euthanized at 8 h post-challenge with an overdose of isoflurane, and the left ventricular puncture drew blood.

### Serum Preparation

Blood from both humans and mice was allowed to clot at room temperature for 30 min and stored at 4°C for 2 h. After centrifugation at 1,600 *g* for 10 min, serum was transferred to a new tube. Samples with visible hemolysis (reddish) were discarded at this step. Next, clear serum was centrifugated at 20,000 *g* for another 10 min at 4°C to remove cell debris and apoptotic bodies. Then, the supernatant was aliquoted and stored at −80° until analysis.

### Serum Small Extracellular Vesicles Isolation

Before sEVs isolation, stored serum was thawed on ice and centrifuged at 21,000 *g* for 15 min at 4°C, and the supernatant was transferred to a new tube. According to the recommended protocol, 63 μL ExoQuick Solution (System Biosciences Inc., Mountain View, CA, United States) was added to every 250 μL serum and incubated at 4°C for 40 min after a brief up and down. After that, the ExoQuick/serum mixture was centrifuged at 1,500 *g* for 30 min, and the supernatant was removed by aspiration. Another 5 min centrifugation was performed to remove the residue liquid. Finally, the sEVs-containing pellet was resuspended in PBS.

### Nanoparticle-Tracking Analysis

Size distribution and concentration of the isolated particles were measured by a Nanosight LM20 (NanoSight, Amesbury, United Kingdom), equipped with a 640-nm laser, and the software used for capturing and analyzing the data was Version 2.3 Build 0034. The instrument was routinely calibrated by 100 nm polystyrene latex standards and particle-free PBS. Before analysis, the isolated sEVs were homogenized. The measurement time was 60 s, and the Frames Per Second was 25. Three measurements were performed for each sample.

### Transmission Electron Microscopy

Transmission electron microscopy (TEM) analysis on isolated particles was performed as described by Thery et al. (2006). Briefly, the pellets dissolved in PBS were mixed with an equal volume of 4% paraformaldehyde and were transferred onto Formvar/carbon-coated nickel grids. After staining with 4% w/v Uranyl Acetate, the morphology and size of sEVs were observed by a TEM (JEM-2100, JEOL, Tokyo, Japan).

### SDS-PAGE and Western Blotting

Total proteins from dissolved pellets were extracted by RIPA lysis buffer (Pierce, Rockford, IL, United States). Protein concentrations were determined using a BCA protein assay kit (Pierce, Rockford, IL, United States). Proteins separated on 12% sodium dodecyl sulfate-polyacrylamide gel electrophoresis were stained by G250 or transferred onto PVDF membranes (Millipore, Bedford, MA, United States). After blocking for 1 h at room temperature with 5% bovine serum albumin, membranes were incubated overnight with antibodies against CD9, CD63, and CD81 (Abcam, Cambridge, MA, United States). After three times washing, the membrane was incubated with HRP-conjugated secondary antibodies and detected by enhanced chemiluminescence (Pierce, Rockford, IL, United States).

### Exosomal RNA Extraction and Quantitation

The serum sEVs precipitated by Exoquick were resuspended in PBS. Then, total RNA was extracted using Trizol LS with the addition of 4 μg glycogen (Invitrogen, Life Technologies, CA, United States) as a carrier and overnight precipitation at −20°C. The RNA quantity was determined using NanoDrop (Thermo Fisher Scientific, Wilmington, DE, United States). The Agilent Bioanalyzer 2100 system assessed the RNA integrity with a small RNA chip (Agilent Technologies, Santa Clara, CA, United States).

### Small RNA Library Construction and Deep Sequencing

The NEB Next^®^ Small RNA Library Prep Set for Illumina^®^ (NEB, Ipswich, MA, United States) was adopted to convert exosomal small RNA into cDNA libraries for next-generation sequencing. According to the manufacturer’s recommendations, total exosomal RNA was ligated with 3′ and 5′ SR adaptors. Then small RNA molecules were reverse transcribed into cDNA, amplified using the adaptor primers for 14 cycles. The amplified libraries were resolved on a native 6% PAGE-gel. DNA fragments between 140 and 160 bp were recovered. Library quality was assessed on the Agilent 2100 Bioanalyzer system. Finally, the purified cDNA was used for cluster generation and sequenced using an Illumina HiSeq 2500 platform, and 50 bp single-end reads were generated. The sequence data were deposited in the National Center for Biotechnology Information (human^2^; mouse^3^).

### Small RNA Sequencing Data Analysis

The small RNA sequencing data were processed as previously described with modification (Zhou et al., 2017). Briefly, clean reads were obtained by removing adapters, reads containing ploy-N, low-quality reads, and sequences smaller than 18 nt. Then, these clean reads were mapped to human and mouse reference genomes (hg38 and mm9) by hisat2 (Kim et al., 2015). The known miRNAs were identified based on miRBase 22.1. Next, DESeq2 software (Love et al., 2014) was used to normalize counts and calculate the differential expression of microRNAs. The differentially expressed miRNAs were selected as follows: | Log2Fold change| ≥ 1 and *p*-value < 0.01.

### Quantitative Reverse Transcriptase Real-Time Polymerase Chain Reaction for Serum Exosomal miRNA

The serum exosomal RNA for the qPCR assay was extracted from 500 μL human serum and 250 μL mouse serum. The procedures were the same as for RNA sequencing. The qRT-PCR was performed using MiDETECTA Track™ miRNA qRT-PCR kits (Riobio Inc., Guangzhou, China) and a CFX 96 PCR system (Bio-Rad, Hercules, CA, United States) following the manufacturer’s protocols. The *Ct* values were normalized by using the spiked 100 fmol synthetic cel-miR-39.

### Data Analyses

Data are presented as the mean ± SEM. A comparison between means was assessed by unpaired Student’s *t*-test or one-way analysis of variance using GraphPad Prism software (Version 5.01). *P* < 0.05 was considered statistically significant.

## Results

### Isolation and Characterization of Small Extracellular Vesicles From Human and Mouse Serum

**FIGURE 1:**
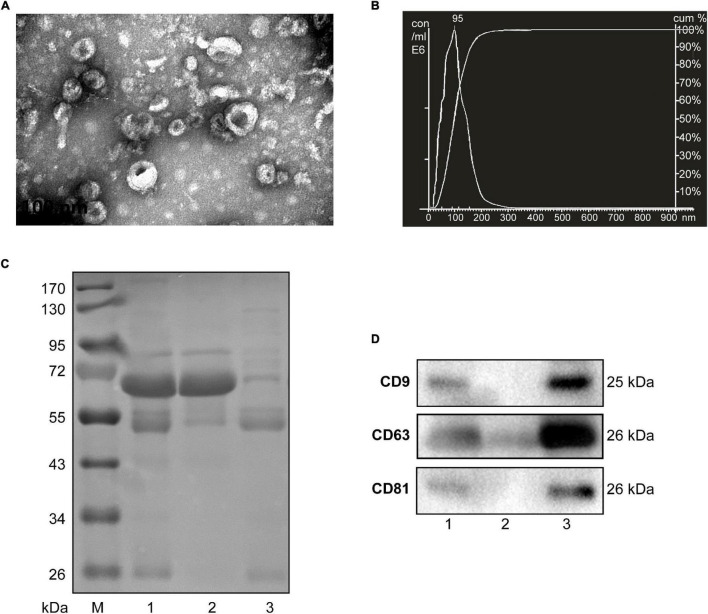
Characterization of sEVs isolated from human serum. **(A)** The TEM image shows the spherical morphology of sEVs with a diameter of approximately 100 nm, bar = 100 nm. **(B)** NTA analysis plot illustrates the size distribution and concentration of the sEVs isolated human serum. **(C)** Proteins from serum, sEVs-depleted serum, and sEVs were separated by SDS-PAGE and stained by Coomassie blue. 40 μg proteins from serum and sEVs depleted serum were loaded. The amount of sEVs protein was adjusted by the serum volume corresponding to 40 μg serum protein. **(D)** The expression of CD9, CD81, and CD63, markers of exosomes, was detected by western blotting. Lane 1: total serum; lane 2: sEVs-depleted serum; lane 3: serum sEVs.

**FIGURE 2:**
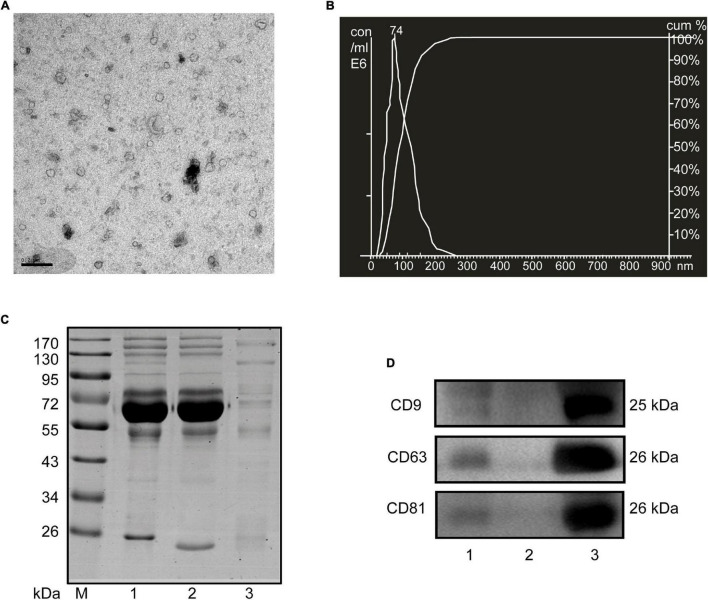
Characterization of sEVs isolated from mouse serum. **(A)** The TEM image depicts the spherical morphology of the isolated sEVs, bar = 200 nm. **(B)** NTA analysis plot illustrates the size distribution and concentration of the sEVs isolated mouse serum. **(C)** Proteins from serum, sEVs-depleted serum, and sEVs were separated by SDS-PAGE and stained by Coomassie blue. 40 μg proteins from serum and sEVs depleted serum were loaded. The amount of sEVs protein was adjusted by the serum volume corresponding to 40 μg serum protein. **(D)** The expression of CD9, CD81, and CD63, markers of exosomes, was detected by western blotting. Lane 1: total serum; lane 2: sEVs-depleted serum; lane 3: serum sEVs.

Considering the limited volume of human and mouse serum and the low yield of sEVs by ultracentrifugation, we chose ExoQuick, a method based on precipitation, to isolate serum sEVs as we did before (Ji et al., 2016; Zhou et al., 2017). TEM analysis showed the spherical vesicles with approximately 100 nm (Figures 1A, 2A). NTA analysis indicated that the size of these isolated EVs ranged from 30 to 150 nm (Figures 1B, 2B). The expression of exosomal markers of CD9, CD63, and CD81 was detected in both human and mouse serum precipitations (Figures 1C,D, 2C,D). Notably, there were no or barely detectable bands in the sEVs-depleted serum, indicating the high serum sEVs recovery efficiency. Calnexin, a negative marker of exosomes, was not detected in sEVs from both human and mouse serum (Supplementary Figure 1). In short, particles obtained from human and C57BL/6 mouse serum by ExoQuick complied with the characteristics of sEVs (Lötvall et al., 2014).

### Differential Serum Small Extracellular Vesicles miRNAs Between Ischemic Stroke Patients and Healthy Controls

**FIGURE 3:**
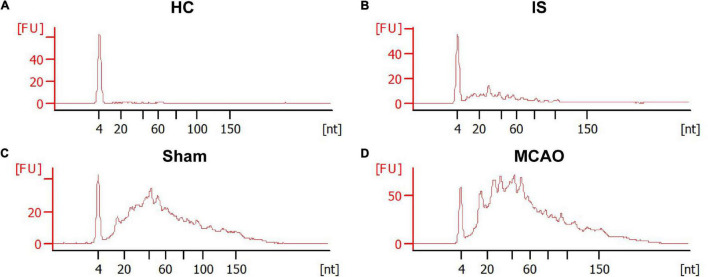
The size distribution and relative abundance of sEVs RNA from IS patients and healthy controls, and tMCAO mice and sham controls. The electropherograms delineate the size distribution in nucleotides (nt) and fluorescence intensity (FU) of small RNA in serum sEVs. The representative electropherograms of HCs **(A)** and IS patients **(B)**; sham **(C)** and tMCAO mice **(D)**.

The RNA content is very low in human serum exosomes (Zhao et al., 2020). To obtain enough exosomal RNA for small RNA sequencing, we extract sEVs from 2 mL of human serum. Therefore, in this study, 40 IS patients and 33 HCs were randomly divided into groups: group 1 included 20 IS patients and 17 HCs; group 2 included 20 IS patients and 16 HCs. 200 μL serum from each person was pooled. The remaining sample of each person was kept for subsequent PCR verification. Bioanalyzer analysis of the exosomal RNA revealed that ischemic stroke significantly increased the amount of serum exosomal RNA [29 ± 11 ng/mL (HCs) vs. 112 ± 26 ng/mL (IS), *p* < 0.05] (Figures 3A,B). The statistical summary of small RNA sequencing and categories of small RNA was provided in Supplementary Tables 1, 2.

In this study, 1,084 and 990 known miRNAs were identified in the two HC groups, and 1,113 and 1,021 were identified in the two IS patient groups (Supplementary Table 5). These analyses identified 1,444 miRNAs, accounting for 54.2% of known human miRNAs (miRbase Release 22.1, 2,656 mature miRNAs, *Homo sapiens*). Differential expression analysis revealed that 208 and 216 miRNAs were up- and downregulated in IS patients, respectively (Supplementary Tables 6, 7), accounting for 29.4% (424/1,440) of the total identified miRNAs. Notably, these differentials were low abundance miRNAs, as the sum read counts of these 424 differential miRNAs accounted for only less than 1% of the total identified. Considering the technical challenge of PCR analysis of these low abundance miRNAs, we focused on the top 25 overexpressed miRNAs, whose read counts accounted for over 85% of the upregulated in IS patients (Table 2). In the downregulated, miR-122-5p was the most abundant miRNA, whose read counts accounted for over 10% of those downregulated.

**TABLE 2:** List of top 25 upregulated serum exosomal miRNAs in IS patients.

			HCs		IS		Tissue specificity	
miRNA species	Log_2_FC	*P* value	1	2	1	2	Tissue	References
hsa-miR-9-3p	7.15	4.41E-37	3.35	10.49	939.79	804.15	brain	Landgraf et al., 2007; Guo et al., 2014; Ludwig et al., 2016
hsa-miR-450b-5p	4.15	5.58E-91	82.04	77.91	1,473.48	1,377.38	n/a	
hsa-miR-124-3p	3.62	2.55E-118	430.27	422.51	5,631.87	4,819.10	brain	Landgraf et al., 2007; Guo et al., 2014; Ludwig et al., 2016
hsa-miR-143-3p	2.42	1.05E-05	13,541.80	15,502.34	37,232.75	117,740.19	brain	Landgraf et al., 2007; Guo et al., 2014
hsa-miR-30e-3p	2.18	3.04E-05	168.26	254.70	622.18	1,285.71	n/a	
hsa-miR-223-3p	2.15	1.92E-92	2,664.50	2,445.14	10,988.66	11,711.82	spleen	Guo et al., 2014
hsa-miR-340-5p	2.08	2.00E-07	190.86	305.64	862.27	1,235.82	n/a	
hsa-miR-4286	2.01	1.44E-27	358.28	361.08	1,564.71	1,337.93	n/a	
hsa-miR-450a-5p	2.00	1.17E-21	180.81	188.78	775.84	697.39	n/a	
hsa-miR-718	1.92	1.69E-28	226.85	233.73	862.96	878.42	n/a	
hsa-miR-223-5p	1.66	2.57E-28	439.48	480.94	1,417.91	1,479.50	spleen	Guo et al., 2014
hsa-miR-221-5p	1.58	7.62E-13	282.10	239.72	819.06	741.49	n/a	
hsa-miR-144-5p	1.54	1.89E-05	282.94	371.56	753.89	1,141.82	n/a	
hsa-let-7a-3p	1.36	5.32E-19	782.69	720.66	2,040.78	1,831.10	n/a	
hsa-miR-98-5p	1.30	1.67E-06	246.11	305.64	613.26	746.13	brain	Landgraf et al., 2007
hsa-miR-338-5p	1.29	1.32E-15	2,724.77	2,404.68	6,778.13	5,754.38	n/a	
hsa-miR-4448	1.25	9.67E-42	3,867.41	3,628.75	9,155.73	8,714.53	n/a	
hsa-miR-32-5p	1.25	3.39E-12	292.15	275.68	674.32	679.99	n/a	
hsa-miR-532-5p	1.20	2.17E-03	549.98	617.28	953.51	1,722.02	n/a	
hsa-miR-93-5p	1.15	7.22E-05	1,380.38	1,627.09	2,648.56	4,020.75	brain	Guo et al., 2014
hsa-miR-193a-5p	1.14	2.66E-09	909.93	807.55	2,062.05	1,720.86	muscle	
hsa-miR-425-3p	1.11	1.94E-08	615.27	575.33	1,402.14	1,162.71	n/a	
hsa-miR-24-3p	1.10	5.70E-13	1,405.49	1,276.50	3,067.69	2,688.62	n/a	
hsa-miR-374a-5p	1.06	8.23E-11	2,993.48	2,662.38	6,431.72	5,396.98	n/a	
hsa-miR-4443	1.01	2.09E-06	738.32	644.25	1,532.47	1,254.38	n/a	

*n/a, not available.*

### Differential Serum Small Extracellular Vesicles miRNAs Between Transient Middle Cerebral Artery Occlusion and Sham Mice

**FIGURE 4:**
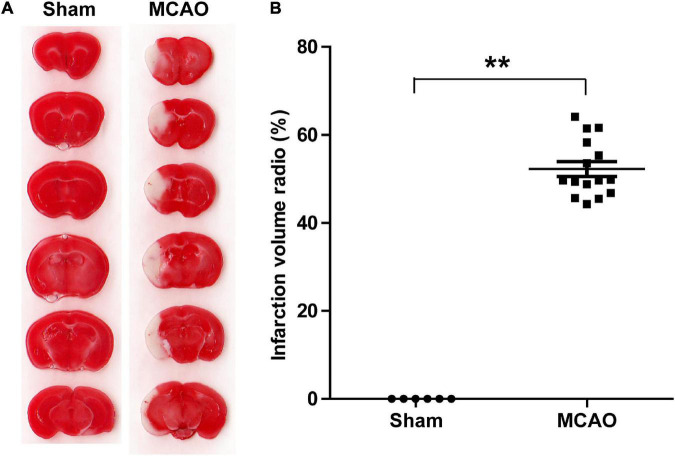
A tMCAO mouse model. **(A)** A representative image of TTC staining, normal tissues were stained in red, while the infarction area was stained in white. The infarct volume was measured by TTC staining 24 h after the onset of ischemia. **(B)** The statistical analysis of infarct volume. The infarct volume was 51.24 ± 7.89% in the tMCAO group; sham group, *n* = 6, tMCAO group, *n* = 15, ** *P* < 0.01.

To determine the effects of acute ischemia on the miRNA profile of mouse serum sEVs, a 60 min tMCAO mouse model was established. The infarct volume in tMCAO mice was around 50% (Figure 4), consistent with the typical characteristics of this IS animal model. Moreover, serum from 7 to 9 tMCAO mice and the corresponding sham mice was pooled, and 500 μL serum was used for small RNA sequencing. Similar to the observation in IS patients, compared with sham controls, the RNA content increased in the serum sEVs from tMCAO mice (Figures 3C,D); however, there was no statistical difference (914.5 ± 3.1 vs. 1,539.0 ± 205.3 ng/mL, *p* = 0.0932).

The statistical summary of small RNA seq and categories of small RNA was provided in Supplementary Tables 3, 4. In the two independent experiments, 739 and 864 known miRNAs were identified from the sham group, and 1,038 and 1,074 were identified from the tMCAO groups (Supplementary Table 8). Thus, 1,373 known miRNAs were identified in these analyses, which accounted for 71.7% of known miRNAs (miRbase Release 22.1, 1,978 mature miRNAs, *Mus musculus*). Compared with the sham group, 20 miRNAs were upregulated in the tMCAO group (Table 3), while 17 were downregulated (Supplementary Tables 9, 10). In line with the finding in IS patients, miR-122-5p was also the most abundant downregulated miRNA.

**TABLE 3:** List of 20 upregulated serum exosomal miRNAs in tMCAO mice.

miRNA species	Fold change	*p*-value	Sham 1	Sham 2	tMCAO 1	tMCAO 2	Tissue	References
mmu-miR-124-3p	7.76	3.89E-06	0.00	0.00	78.19	59.71	brain	Lagos-Quintana et al., 2002; Landgraf et al., 2007; Guo et al., 2014; Ludwig et al., 2016
mmu-miR-9-3p	7.05	2.48E-09	1.79	1.66	261.41	194.77	brain	Lagos-Quintana et al., 2002; Landgraf et al., 2007; Guo et al., 2014; Ludwig et al., 2016
mmu-miR-668-3p	6.45	6.73E-04	0.00	0.00	31.46	24.17	n/a	
mmu-miR-770-3p	6.37	8.79E-04	0.00	0.00	29.15	23.46	n/a	
mmu-miR-9-5p	6.12	7.09E-14	12.56	1.66	547.34	427.93	brain	Lagos-Quintana et al., 2002; Guo et al., 2014
mmu-miR-323-3p	5.57	1.24E-03	0.00	1.66	43.95	37.67	brain	Guo et al., 2014; Ludwig et al., 2016
mmu-miR-219b-5p	5.39	2.03E-03	1.79	0.00	39.33	33.41	brain	Liang et al., 2007; Ludwig et al., 2016
mmu-miR-341-3p	4.98	6.87E-03	0.00	1.66	34.24	19.90	n/a	
mmu-miR-129b-3p	4.49	1.23E-08	8.97	4.99	158.69	152.83	brain	Liang et al., 2007; Guo et al., 2014; Ludwig et al., 2016
mmu-miR-129-5p	3.82	6.73E-08	8.97	21.64	189.23	244.53	brain	Guo et al., 2014; Ludwig et al., 2016
mmu-miR-433-3p	3.43	2.40E-06	8.97	16.64	151.29	125.11	brain	Ludwig et al., 2016
mmu-miR-598-3p	3.14	2.20E-03	5.38	4.99	54.13	36.96	n/a	
mmu-miR-300-3p	2.41	3.79E-03	17.94	8.32	78.65	60.42	n/a	
mmu-miR-434-3p	2.25	1.06E-04	35.88	34.95	184.60	152.12	n/a	
mmu-miR-107-3p	1.66	2.73E-03	145.30	83.22	397.89	321.30	n/a	
mmu-miR-382-5p	1.61	5.70E-03	48.43	51.60	177.66	127.95	n/a	
mmu-miR-128-3p	1.34	1.59E-03	6,709.11	9,163.98	20,672.90	19,481.50	brain	Lagos-Quintana et al., 2002; Guo et al., 2014; Ludwig et al., 2016
mmu-let-7d-5p	1.31	1.54E-03	726.52	675.73	1,889.07	1,580.93	n/a	
mmu-miR-423-5p	1.12	6.68E-03	1,462.01	1,266.58	2,693.65	3,234.36	n/a	
mmu-miR-328-3p	1.08	6.85E-03	832.36	888.77	1,905.73	1,738.02	n/a	

*n/a, not available.*

### Acute Ischemia Increases a Panel of Brain-Specific miRNAs in Serum Small Extracellular Vesicles From Both Ischemic Stroke Patients and Transient Middle Cerebral Artery Occlusion Mice

In addition to stability, tissue specificity is another essential trait of circulating miRNAs as biomarkers. Based on the tissue-specific or enriched miRNAs information provided by these previous studies (Landgraf et al., 2007; Liang et al., 2007; Ludwig et al., 2016), we were excited to find that five brain-specific miRNAs were included in the top 25 upregulated miRNAs in IS patients: hsa-miR-9-3p, hsa-miR-124-3p, hsa-miR-143-3p, hsa-miR-98-5p, and hsa-miR-93-5p (Table 2). Likewise, 9 out of the 20 upregulated miRNAs in tMCAO mice are brain-specific or preferentially expressed in brain tissues, including mmu-miR-124-3p, mmu-miR-9-3p, mmu-miR-9-5p, mmu-miR-323-3p, mmu-miR-219b-5p, mmu-miR-129b-3p, mmu-miR-129-5p, mmu-miR-433-3p, and mmu-miR-128-3p (Table 3). The coordinate upregulation of a set of brain-specific miRNAs in serum sEVs from IS patients, and tMCAO mice suggested a conservative regulatory mechanism.

### Validation of the Brain-Specific miRNAs Identified in Ischemic Stroke Patients

**FIGURE 5:**
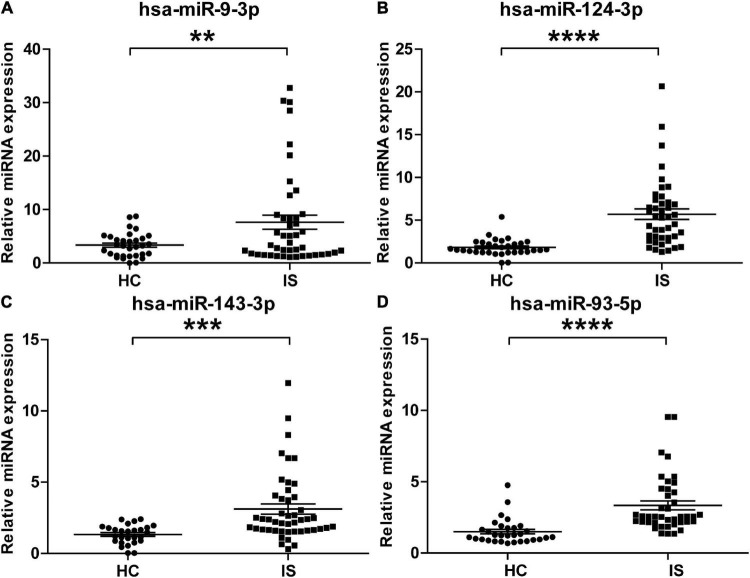
Validation of the upregulation of the four brain-specific miRNAs in stroke patients. The expression of hsa-miR-9-3p **(A)**, hsa-miR-124-3p **(B)**, hsa-miR-143-3p **(C)**, and hsa-miR-93-5p **(D)** was assessed using qRT-PCR in the individual samples for small RNA sequencing. IS patients, *n* = 40, healthy controls (HCs), *n* = 33. The *Ct* value of target miRNAs was normalized against Cel-mir-39 and compared to the mean of healthy controls. Data are presented as Mean ± SEM. Unpaired Student’s test vs. healthy control (**P < 0.01, ***P < 0.001, ****P < 0.0001).

To validate the overexpressed brain-specific miRNAs identified by small RNAseq, the relative expression of 4 brain-specific miRNAs (hsa-miR-9-3p, hsa-miR-124-3p, hsa-miR-143-3p, and hsa-miR-93-5p) was determined by using qRT-PCR in the individual serum samples for RNAseq analysis. Consistent with the tendency of the RNAseq results, the levels of all four miRNAs significantly increased in serum sEVs from IS patients, and miR-9-3p was the most significantly modulated (Figure 5).

### Temporal Expression of Serum Small Extracellular Vesicles miR-124-3p, miR-9-5p, miR-129-5p, and miR-433-3p in Transient Middle Cerebral Artery Occlusion Mice

**FIGURE 6:**
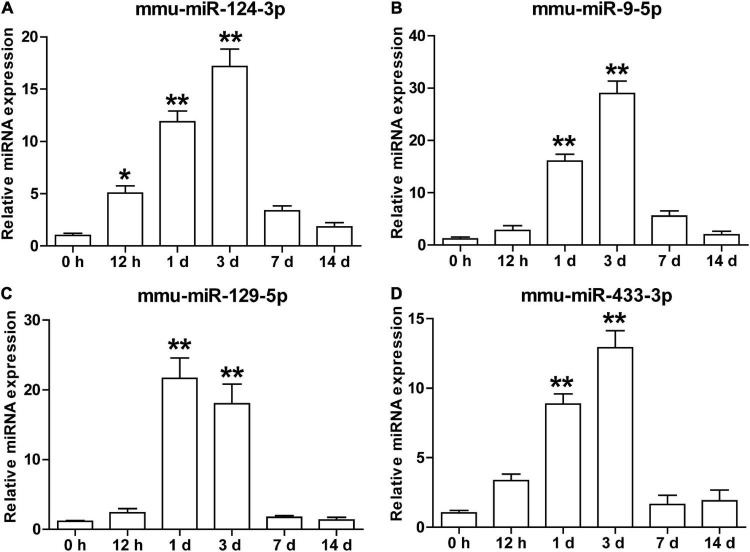
The temporal expression of brain-specific miRNAs in the serum sEVs from the tMCAO mouse model. The levels of mmu-miR-124-3p **(A)**, mmu-miR-9-5p **(B)**, mmu-miR-129-5p **(C)**, and mmu-miR-433-3p **(D)** in mouse serum sEVs were detected by qRT-PCR at 0.5, 1, 3, 7, and 14 days after the onset of ischemia. The *Ct* value was normalized against mmu-miR-451. Each time point represents the mean of 3 pooled serum samples (4–6 animals per sample). Data were presented as Mean ± SEM. Statistical significance was determined by non-parametric one-way ANOVA followed by Dunn’s post-test, *p < 0.05, **p < 0.01 vs. sham control (0 h).

To explore the potential biological significance of the increament of these brain-specific miRNAs in the serum sEVs after cerebral ischemia, we examined the temporal dynamics of the mmu-miR-124-3p, mmu-miR-9-5p, mmu-miR-129-5p, and mmu-miR-433-3p in a tMCAO mouse model. As illustrated in Figure 6, these four miRNAs shared a similar expression pattern: their expression started to increase at 0.5 days after ischemia, peaked at day 1 or 3, and returned to normal levels at day 7 and 14 (Figure 6). This temporal expression pattern is coordinated with the evolution of ischemic injury in animal stroke models (Rewell et al., 2017).

### Effects of Lipopolysaccharide Challenge on the Expression of Brain-Specific miRNAs in the Mouse Serum Small Extracellular Vesicles

**FIGURE 7:**
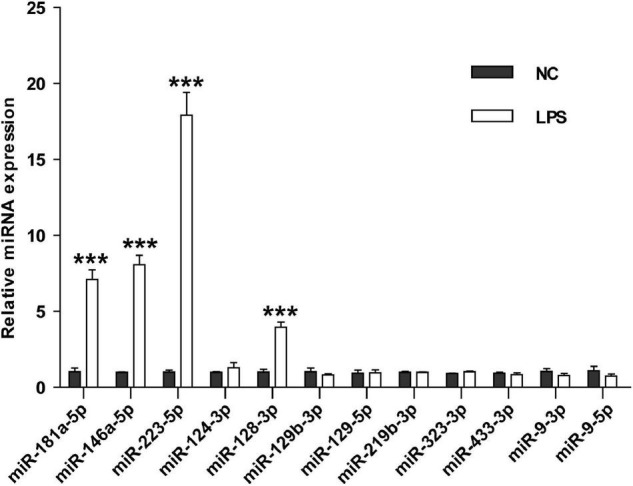
The effects of LPS challenge on the expression of brain-specific miRNAs in the mouse serum sEVs. RT-PCR assessed the expression of nine brain-specific miRNAs and three inflammatory miRNAs in the mouse serum sEVs. Data were expressed as the fold change ± SEM (*n* = 3) compared to normal control (NC). Statistical significance was determined by student *t*-test. ***p < 0.001.

To investigate whether these miRNAs could differentiate cerebral ischemia injury from other neurological and non-neurological diseases, we examined the levels of the nine miRNAs in the serum sEVs derived from LPS challenged mice, a widely used animal model of neuroinflammation. Three miRNAs involved in inflammation, miRNA-181a-5p, miRNA-146a-5p, and miRNA-223-5p (Xie et al., 2013; Ferreira et al., 2015), were included as positive controls. Compared with the vehicle controls, the expression of these three inflammatory miRNAs significantly increased in the serum sEVs from LPS treated mice, especially miR-223-5p. However, except for miR-128-3p, LPS treatment did not significantly affect the levels of the other eight miRNAs (Figure 7).

## Discussion

By comprehensively analyzing the effects of acute cerebral ischemia on the serum sEVs miRNA profile of IS patients and tMCAO mice, this study revealed a significant increase in brain-specific miRNAs in the serum sEVs from both IS patients and tMCAO mice. Further animal studies indicated that the temporal expression of these brain-specific miRNAs in serum sEVs was closely correlated with the evolution of ischemic brain injury and recovery, and the expression of these miRNAs was unaffected by neuroinflammation.

A challenge in screening serum miRNAs for IS diagnosis is the poor reproducibility, as the candidate serum miRNAs identified in different studies can hardly be verified against each other (Eyileten et al., 2018). However, this situation has been greatly changed in the studies based on serum sEVs. The differential miRNAs identified in the present RNAseq study were well corroborated by these previous reports, such as the upregulation of hsa-miR-9 and hsa-miR-124 (Ji et al., 2016), hsa-miR-223 (Chen et al., 2017), and hsa-miR-450b-5p (Luo et al., 2019), as well as the downregulation of miR-422a and miR-125b-2-3 (Li D.B. et al., 2017). Our data also showed the overexpression of miRNA-21-3p and miRNA-30a-5p (Wang et al., 2018), but they did not reach the significance threshold. In a latest microarray study, four upregulated miRNAs (hsa-miR-17-5p, hsa-miR-20b-5p, hsa-miR-93-5p, hsa-miR-27b-3p) were identified in serum exosomes of IS patients (Van Kralingen et al., 2019), among which two (hsa-mir-20b-5p and hsa-mir-93-5p) were also included in our list (Table 2 and Supplementary Table 7). The cross-validation of results from different studies highlights the advantages of using sEVs miRNAs for IS diagnosis and prognosis.

This advantage could be related to the features of sEVs miRNAs. Circulating miRNAs mainly exist in two forms, binding with protein/lipoprotein or packaged in membrane vesicles (Creemers et al., 2012). A recent study evidenced the different effects of cerebral ischemia on the different forms of circulating miRNA. In the three potential biomarkers for acute IS (miR-125a-5p, miR-125b-5p, and miR-143-3p), only a significant upregulation of miR-143-3p was observed in vesicles from IS patients (Tiedt et al., 2017). Our data consistently showed the overexpression of miR-143-3p in the serum sEVs of IS patients (Table 2 and Figure 5). The other two were not detected in this study, probably because of their low abundance in serum sEVs. Therefore, sEVs isolation could reduce the complexity of the samples by removing the protein/lipoprotein-binding RNA and produce a more stable result.

A survey on tumor biomarker studies emphasized the importance of tissue specificity of biomarkers. They argued that many potential miRNA biomarkers for cancer were those highly expressed in blood cells, which reflected a blood cell-based phenomenon rather than a cancer-specific origin. The most exciting finding of this study is the coordinate upregulation of a panel of brain-specific miRNAs in the serum sEVs from both IS patients (Table 2) and tMCAO mice (Table 3). miR-9 and miR-124 were the two significantly upregulated miRNAs shared by the IS patients and the tMCAO mice. Moreover, previous studies have proposed that these two miRNAs could be diagnosis and prognosis markers of IS (Ji et al., 2016; Rainer et al., 2016; He et al., 2019). The specificity and sensitivity of these brain-specific miRNAs in the diagnosis and prognosis of ischemic stroke need to be further evaluated in large-scale clinical studies.

An important issue for the translational application of circulating miRNAs in the IS diagnosis is the biological significance they reflect. Several recent biomarker studies on IS began to look at this issue. For instance, the elevated circulating miR-125a-5p, miR-125b-5p, and miR-143-3p may indicate the thrombotic processes (Tiedt et al., 2017), while the changes of miRNA-17 family members in sEVs could reflect the development of cerebrovascular disease (Van Kralingen et al., 2019).

The blood-brain barrier (BBB) is a strictly modulated interface between the peripheral circulation and the central nervous system (CNS) (Pardridge, 2003). Due to the nanoscale and the lack of specific markers, whether and when sEVs released by brain cells can cross the BBB remains uncertain (Shi et al., 2019). Our temporal analysis indicated that the dynamics of these brain-specific miRNAs were closely correlated with the progression of ischemic brain injury and recovery. These temporal dynamics implicated that sEVs in the brain could hardly cross the intact BBB, and the increament of these brain-specific miRNAs in serum sEVs could be closely associated with BBB disruption. Consistently, two recent studies also showed that sEVs could not cross intact BBB in normal conditions (Ridder et al., 2014; Chen et al., 2016).

However, in contrast to the reports that exosomes can cross BBB after LPS treatment (Ridder et al., 2014; Chen et al., 2016), our data suggested that sEVs harboring brain-specific miRNA could hardly cross the BBB under inflammatory circumstances because the expression of eight brain-specific miRNAs in the serum sEVs was not significantly affected by LPS treatment. This discrepancy may be due to the discrepancies in the tracking methods and experimental models. In a study, luciferase (Chen et al., 2016) was used to track sEVs in an *in vitro* BBB model (Chen et al., 2016). In another study, a transgenic mouse expressing Cre recombinase specifically in the hematopoietic lineage was used to track blood sEVs (Ridder et al., 2014). More efforts and novel tracking methods are needed to provide direct evidence for the connections between the increased brain-specific miRNAs in serum sEVs and the compromise of BBB after cerebral ischemia.

## Conclusion

In this study, we took a comprehensive view of the impact of acute ischemia on the serum sEVs miRNA profiles of ischemic stroke (IS) patients and MCAO mice for the first time. More importantly, this study demonstrated the increase of a set of brain-specific miRNAs in the serum sEVs after acute cerebral ischemia and suggested that these increased brain-specific miRNAs in serum sEVs could be promising biomarkers that directly reflect the ischemic brain injury.

## Data Availability Statement

The datasets presented in this study can be found in online repositories. The names of the repository/repositories and accession number(s) can be found below: https://www.ncbi.nlm.nih.gov/bioproject/PRJNA607025; mouse, https://www.ncbi.nlm.nih.gov/bioproject/PRJNA607346.

## Ethics Statement

The studies involving human participants were reviewed and approved by the Ethics Committee of Affiliated Hospital of Nantong University. The patients/participants provided their written informed consent to participate in this study. The animal study was reviewed and approved by the Animal Experimental Committee of Jinan University.

## Author Contributions

YJ and QJ conceived and supervised the study. MS collected blood samples and clinical data. XZ, CX, SL, DC, YP, and YD performed experiments. ZC did the RNAseq data analysis. YJ, XZ, and QJ drafted the manuscript. JJ reviewed and revised the manuscript. All authors contributed to the article and approved the submitted version.

## Conflict of Interest

The authors declare that the research was conducted in the absence of any commercial or financial relationships that could be construed as a potential conflict of interest.

## Publisher’s Note

All claims expressed in this article are solely those of the authors and do not necessarily represent those of their affiliated organizations, or those of the publisher, the editors and the reviewers. Any product that may be evaluated in this article, or claim that may be made by its manufacturer, is not guaranteed or endorsed by the publisher.

## Footnotes

1
http://imagej.nih.gov/ij/

2
https://www.ncbi.nlm.nih.gov/bioproject/PRJNA607025

3
https://www.ncbi.nlm.nih.gov/bioproject/PRJNA607346

## Supplementary Material

Supplementary Figure 1 The expression of Calnexin, a negative marker of sEVs, was detected by western blotting. Calnexin was not detected in sEVs from both human and mouse serum (de-sEV: sEVs-depleted serum).

Supplementary Table 1 Summary of small RNA-Seq analysis of human serum sEVs.

Supplementary Table 2 Summary of small RNA classification in human serum sEVs.

Supplementary Table 3 Summary of small RNA-Seq analysis of mouse sEVs.

Supplementary Table 4 Summary of small RNA classification in mouse sEVs.

Supplementary Table 5 List of miRNAs detected in the serum sEVs from IS patients and healthy controls.

Supplementary Table 6 List of the normalized read counts of miRNAs detected in the serum sEVs from IS patients and healthy controls.

Supplementary Table 7 List of differential serum sEVs miRNAs between IS patients and healthy controls.

Supplementary Table 8 List of miRNAs detected in the serum sEVs from tMCAO and sham mice.

Supplementary Table 9 List of the normalized read counts of miRNAs detected in the serum sEVs from tMCAO and sham mice.

Supplementary Table 10 List of differential serum sEVs miRNAs between tMCAO and sham mice.

## Abbreviations

sEVs: small extracellular vesicles
IS: ischemic stroke
tMCAO: transient middle cerebral artery occlusion
CT: Computed Tomography
MRI: Magnetic Resonance Imaging
HCs: healthy controls
HDL: high-density lipoprotein
LDL: low-density lipoprotein
n/a: not available
RBC: red blood cell
TIA: transient ischemic attack
WBC: white blood cell
TTC: 2,3,5-triphenyltetrazolium chloride
LPS: lipopolysaccharide
PBS: phosphate-buffered saline
NTA: nanoparticle-tracking analysis
TEM: transmission electron microscopy
qRT-PCR: quantitative reverse transcriptase real-time polymerase chain reaction
nt: nucleotides
FU: fluorescence intensity
BBB: blood-brain barrier
CNS: central nervous system
SDS-PAGE: sodium dodecyl sulfate polyacrylamide gel electrophoresis.
