# Supplementary figures and images for: Acute Cerebral Ischemia Increases a Set of Brain-Specific miRNAs in Serum Small Extracellular Vesicles
Source: Front Mol Neurosci. 2022 Apr 27;15:874903. doi: 10.3389/fnmol.2022.874903 (PMC9094043; doi:10.3389/fnmol.2022.874903)

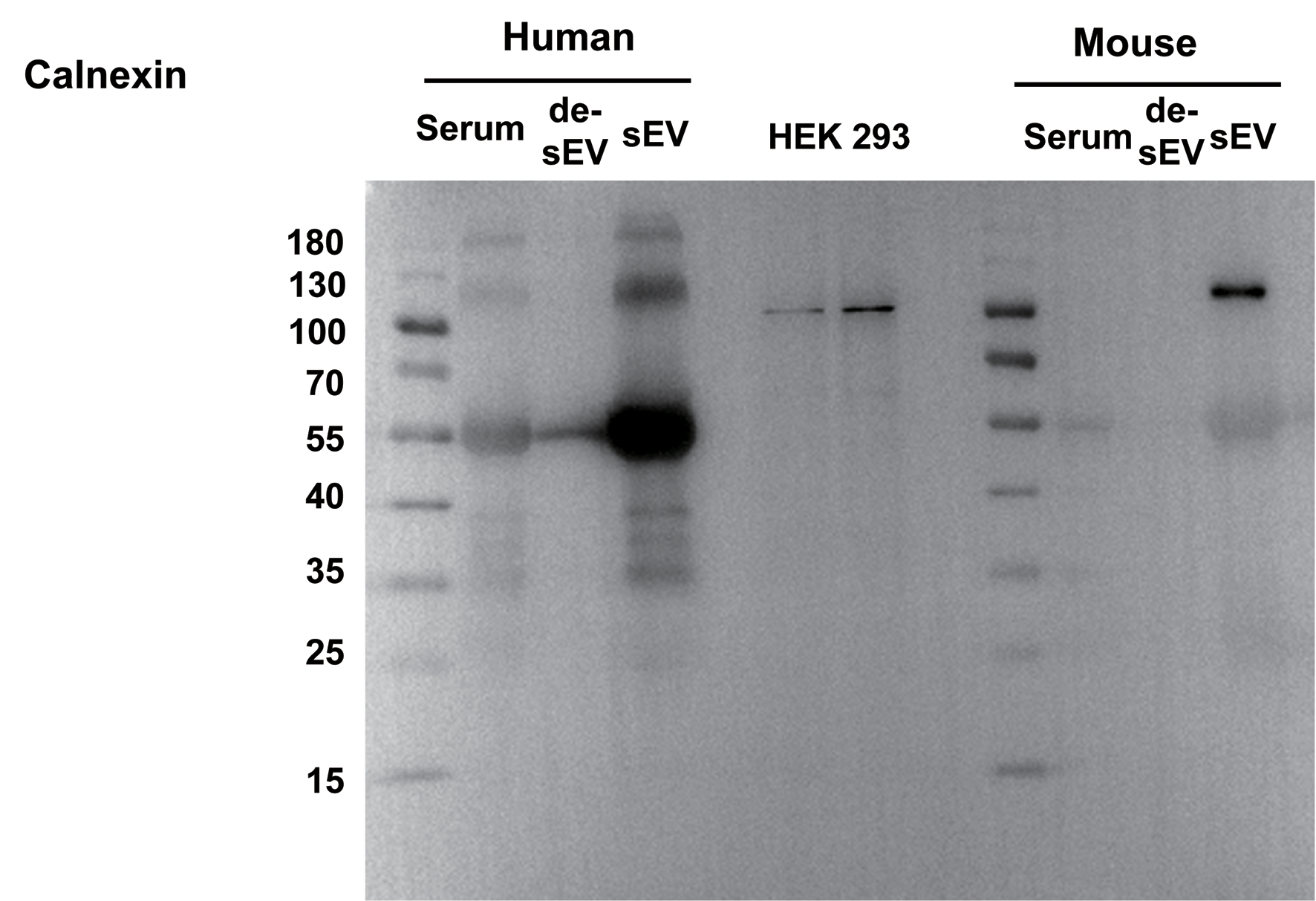

Supplement: Supplementary Figure 1 — The expression of Calnexin, a negative marker of sEVs, was detected by western blotting. Calnexin was not detected in sEVs from both human and mouse serum (de-sEV: sEVs-depleted serum). [file Image_1.tif]
